# Homœopathic Medical Society of the State of New York

**Published:** 1899-08

**Authors:** John L. Moffat

**Affiliations:** Secretary, 17 Schermerhorn street, New York (Brooklyn Borough)


### HOMŒOPATHIC MEDICAL SOCIETY OF THE STATE OF NEW YORK.

The thirty-third semi-annual meeting will be held at Binghamton, N. Y., September 19th and 20th, 1899. The forty-eighth annual meeting will be held at Albany, February 13th and 14th, 1900.

Officers for 1899-1900: President, Jay W. Sheldon, 501 Fayette Park, Syracuse; Vice-Presidents, F. W. Adriance, 306 Lake street, Elmira; George E. Gorham, 160 Hamilton street, Albany; Newton M. Collins, 43 East avenue, Rochester; Secretary, John L. Moffat, 17 Schermerhorn street, New York, B'kn; Treasurer, Charles Deady, 110 W. Forty-eighth street, New York, Mhn; Necrologist, W. S. Garnsey, Gloversville; Counsel, J. C. Coleman, Esq., 100 Broadway, New York, Mhn; Censors, M. O. Terry, Utica; J. E. Slaught, Warsaw; H. D. Schenck, New York; C. E. Teets, New York; L. A. Frazier, Amsterdam; L. A. Martin, Binghamton; M. E. Graham, Rochester; W. L. Hartman, Syracuse; D. M. Hibbard, Olean; Fred. D. Lewis, Buffalo; E. G. Cox, Albany; A. W. Palmer, New York.

The semi-annual meeting will be held as usual on Tuesday and Wednesday, September 19th and 20th. Binghamton, the place of meeting is easy of access, with ample hotel accommodations; the sessions will be held in the Hotel Bennett, which will give our members a discount of $1.00 a day from their regular rate of $4.00. Other hotels at $2.00 a day and upward, are the Arlington, the Crandall, and the Lewis.

Each appointee on a bureau has pledged a paper; papers are to be in the hands of their respective chairmen in time for submission to their disputants; the title must be in the hands of the Secretary two weeks before each meeting for announcement in the programme.

Attention is called to by-law V.

In justice to subsequent bureaus, papers should be presented in abstract if their reading is likely to occupy more than fifteen minutes. It is the duty of chairmen of following bureaus to speak for those interested in their respective bureaus and to object to extensions of time. When the writer of a paper is absent his paper will be read by title unless the Society is ahead of its schedule.

Society dues are a debt of honor!

This anouncement is sent to every homœopathic physician in the State with the hope that he or she will attend the meeting and, if not already a member, join the Society. See the Committee on Increasing Membership. The work of this Society has been of practical benefit to every homœopath in the State; every one who shares these benefits should contribute to the advancement of the Society.

The undersigned appeals to every reader of this for corrections (at any time) to our State Directory, for "good openings" for practice, and for the times and places of meetings and names of officers of our county societies.

JOHN L. MOFFAT,
*Secretary*,
17 Schermerhorn street, New York (Brooklyn Borough).

---

EPILEPSY is often difficult to diagnose. It may be so light that the sufferer is unaware of his infirmity. He may stop in the midst of conversation, be silent a short time, and then continue unconscious that his talk has been interrupted. The subject may have attacks at night and awake in the morning unaware of its occurrence. The objective signs are tremor of the tongue, with sometimes sores from biting during a convulsion, and hemorrhage of the conjunctiva and muscles, particularly abdominal, as also malformations of the ears and skull with protruding under-jaw.—*Charlotte Medical Journal.*

